# Spatiotemporal Regulation and Lineage Specification in Embryonic Endochondral Ossification

**DOI:** 10.3390/ijms27020926

**Published:** 2026-01-16

**Authors:** Sixun Wu, Keita Kondo, Yuki Matsushita

**Affiliations:** 1 Department of Skeletal Development and Regenerative Biology, Nagasaki University Graduate School of Biomedical Sciences, Nagasaki 852-8588, Japan; jj20250315@ms.nagasaki-u.ac.jp (S.W.); bb55325203@ms.nagasaki-u.ac.jp (K.K.); 2 Leading Medical Research Core Unit, Life Science Innovation, Nagasaki University Graduate School of Biomedical Sciences, Nagasaki 852-8523, Japan

**Keywords:** endochondral ossification, mesenchymal condensation, cartilage anlage, lineage tracing, dorsoventral patterning, skeletal disorders

## Abstract

Long bone formation in vertebrates proceeds via endochondral ossification, a sequential process that begins with mesenchymal condensation, advances through cartilage anlage formation, and culminates in its replacement by mineralized bone. Recent advances in inducible lineage tracing and single-cell genomics have revealed that, rather than being a uniform event, mesenchymal condensation rapidly segregates into progenitor pools with distinct fates. Centrally located Sox9^+^/Fgfr3^+^ chondroprogenitors expand into the growth plate and metaphyseal stroma, peripheral Hes1^+^ boundary cells refine condensation via asymmetric division, and outer-layer Dlx5^+^ perichondrial cells generate the bone collar and cortical bone. Concurrently, dorsoventral polarity established by Wnt7a–Lmx1b and En1 ensures that dorsal progenitors retain positional identity throughout development. These lineage divergences integrate with signaling networks, including the Ihh–PTHrP, FGF, BMPs, and WNT/β-catenin networks, which impose temporal control over chondrocyte proliferation, hypertrophy, and vascular invasion. Perturbations in these programs, exemplified by mutations in Fgfr3, Sox9, and Dlx5, underlie region-specific skeletal dysplasias, such as achondroplasia, campomelic dysplasia, and split-hand/foot malformation, demonstrating the lasting impacts of embryonic patterning errors. Based on these insights, regenerative strategies are increasingly drawing upon developmental principles, with organoid cultures recapitulating ossification centers, biomimetic hydrogels engineered for spatiotemporal morphogen delivery, and stem cell- or exosome-based therapies harnessing developmental microRNA networks. By bridging developmental biology with biomaterials science, these approaches provide both a roadmap to unravel skeletal disorders and a blueprint for next-generation therapies to reconstruct functional bones with the precision of the embryonic blueprint.

## 1. Introduction

Embryonic bone development provides a paradigm to advance therapeutic strategies for skeletal disorders, ranging from congenital malformations (e.g., achondroplasia and multiple epiphyseal dysplasia) to post-traumatic regeneration [1,2,3]. Vertebrate long bone morphogenesis predominantly proceeds via endochondral ossification, a hierarchical process encompassing mesenchymal condensation, chondrogenic differentiation into cartilage anlage, and its progressive replacement by mineralized bone [4,5]. In contrast, intramembranous ossification, which accounts for most craniofacial skeletal development, bypasses chondrogenesis via direct osteoblast differentiation [6,7]. The endochondral pathway is clinically relevant because of its spatiotemporally layered regulation of cell fate and causal associations with limb deformities and metabolic bone diseases in preterm infants [8]. Therefore, limb morphogenesis underpins the formation of most appendicular and axial skeletal elements.

In murine development, this program is initiated around embryonic day (E)-10.5 via mesenchymal condensation marked by paired-related homeobox 1 (Prrx1) and SRY-box transcription factor 9 (Sox9) expression [9,10]. Subsequently, this aggregate forms a cartilage anlage (around E12.5) marked by Sox9, with a core of chondrocytes predominantly expressing fibroblast growth factor (Fgf) receptor 3 (Fgfr3), and surrounded by a stratified perichondrium consisting of an inner layer marked by Sp7/osterix (Osx) and an outer layer marked by distal-less homeobox 5 (Dlx5) [10,11]. Prior to the onset of chondrocyte hypertrophy, osteoprogenitors in the perichondrium differentiate to form a circumferential bone collar surrounding the cartilage anlage, providing structural support and signaling cues that constrain hypertrophic differentiation [4]. At the next stage (approximately E15.5), vascular invasion of the hypertrophic zone recruits osteoprogenitors and establishes the primary ossification center [11]. This osteogenic transition is critically dependent on the runt-related transcription factor 2 (Runx2), which serves as a master regulator of osteoblast lineage commitment and differentiation during endochondral ossification [12]. Concomitantly, the anlage exhibits a tripartite zonal architecture (resting, proliferative, and hypertrophic chondrocyte layers) coordinated via Indian hedgehog (Ihh)/parathyroid hormone (PTH)-related protein (PTHrP)-mediated reciprocal signaling [13,14]. Postnatally, coupled bone resorption and formation remodel this anlage into a biomechanically competent structure, with chondrocyte and perichondrial lineages giving rise to distinct skeletal cell types [15,16]. This remodeling ultimately produces lamellar bone, a densely organized cortical structure that imparts the biomechanical strength characteristic of mature long bones [17].

Lineage-tracing studies using constitutive drivers (e.g., *Prrx1-Cre*, *Sox9-Cre*, collagen [*Col*]-*2a1-Cre*, *Col10a1-Cre*, *Osx-Cre*, and *Dermo1-Cre*) have mapped skeletal progenitors and elucidated their developmental dynamics [9,10,18,19]. Moreover, the advent of inducible CreER/loxP systems (e.g., *Sox9-CreER*, *Fgfr3-CreER*, *Dlx5-CreER*, platelet-derived growth factor receptor-alpha [*Pdgfra*]*-CreER*, *Col2a1-CreER*, *Osx-CreER*, Hes family bHLH transcription factor 1 [*Hes1*]*-CreER*, and GLI family zinc finger 1 [*Gli1*]*-CreER*) has resolved previously unrecognized heterogeneity and dorsoventral spatial biases in mesenchymal condensations [16,20,21,22,23,24,25,26]. Contemporary single-cell multi-omics and spatial transcriptomics have further delineated discrete progenitor clusters during embryogenesis, elucidating the mechanisms by which early commitment events program lifelong skeletal architecture [8,27]. These reports highlight the need for fully integrated multiscale approaches to resolve both the heterogeneity of chondrogenic and osteogenic lineages and spatiotemporal patterning. Classical staging, inducible lineage tracing, and single-cell methodologies have shown the ways in which mesenchymal condensation orchestrates growth plate cytoarchitecture and ultimately dictates adult skeletal form and function.

Despite extensive knowledge of signaling pathways governing endochondral ossification, it remains less clear how early mesenchymal condensation events partition progenitor populations in space and time, and how these early decisions pre-pattern dorsoventral growth plate organization and long-term skeletal architecture. Previous studies have largely focused on individual signaling cascades or later stages of growth plate regulation, with limited integration of lineage-based evidence resolving early progenitor heterogeneity.

In this review, we synthesize classical developmental staging with inducible lineage-tracing studies and recent single-cell and spatial approaches to address how mesenchymal condensation gives rise to distinct chondrogenic, perichondrial, and stromal lineages, and how these lineages acquire positional bias during embryogenesis. By framing limb development through the lens of early progenitor specification and spatial patterning, we aim to provide a unified framework linking embryonic condensation dynamics to growth plate organization, skeletal disease, and regenerative potential.

## 2. Specification and Early Fates of Mesenchymal Condensation Progenitors

Mesenchymal condensation, the first visible step in endochondral ossification, depends on a tightly coordinated network of transcription factors to establish skeletal progenitor identity [28]. This process begins around E9.5–10.5, when Prrx1 is broadly expressed across the undifferentiated limb bud mesenchyme [9,29]. As a pioneering transcription factor, Prrx1 primes cells for skeletal commitment and directs them to migrate and aggregate into nascent condensations [10,30]. Once condensation begins at approximately E10.5, Prrx1 expression, which is initially high, is progressively downregulated as Sox9 expression increases, marking the transition from a multipotent mesenchymal state to a committed chondrogenic program [29,31]. At this stage, Sox9 activates cartilage matrix genes, particularly *Col2a1* and aggrecan (*Acan*), thereby contributing to the stabilization of cell–cell adhesion and initiation of cartilage anlage formation [32,33]. Fate mapping with *Sox9-CreER* has revealed that cells labeled at E10.5 populate both the central cartilage core and surrounding perichondrium and that their descendants persist throughout postnatal life without an initial dorsoventral preference [16]. Similarly, *Dermo1-Cre* (*Twist2-Cre*) is active in the lateral plate mesoderm from E9.5 onward and robustly labels the condensed mesenchyme destined to generate both chondrocytes and osteoblasts as well as perichondrial and periosteal cells enveloping the cartilage anlage [18]. Simultaneously, inducible *Pdgfra-CreER*, when activated at E9.5, marks a broad population of multipotent mesenchymal progenitors. Although PDGFRα expression is downregulated in Sox9-positive chondroprogenitors, it persists in the perichondrium, enabling *Pdgfra-CreER* lineage tracing to capture both cartilage and perichondrial osteogenic lineages [21].

**Figure 1:**
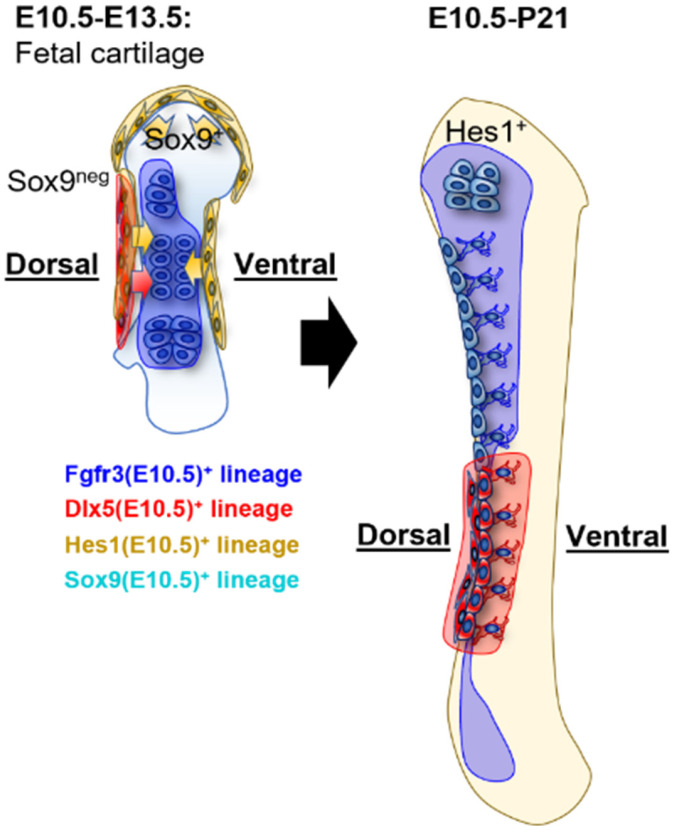
Dorsoventral patterning of limb-bud mesenchymal condensation at E10.5. Tamoxifen was administered at E10.5 to activate CreER drivers. At E13.5, Sox9^+^ descendants (light blue) occupy the cartilage core; Hes1^+^ descendants (yellow) form a rim and begin to invade the perichondrium; Fgfr3^+^ descendants (dark blue) are enriched in the dorsal resting zone; and Dlx5^+^ descendants (red) localize to the outer perichondrial layer. By P21, Sox9^+^ and Hes1^+^ lineages contribute broadly to chondrocytes, osteoblasts, and marrow stromal cells, whereas Fgfr3^+^ and Dlx5^+^ lineages retain a dorsal bias, preferentially contributing to dorsal metaphyseal chondrocytes and to the bone collar, periosteum, and diaphyseal cortex, respectively. To facilitate visualization of lineage contributions in the mature skeleton, an ossified limb schematic at P21 is included using the same color code. This figure is modified from Wu et al. [16].

Concurrent with the emergence of central chondroprogenitors, peri-condensation mesenchyme activates Notch signaling, as previously revealed by the *CBF1:H2B-Venus* reporter [26,34]. Concurrent with the emergence of central chondroprogenitors, peri-condensation mesenchyme activates Notch signaling, which induces Hes1 in cells surrounding Sox9^+^ condensation and, by modulating Sox9 and Runx2 activity, sharpens the condensation boundary and delays premature differentiation [26,35]. Lineage tracing of *Hes1-CreER*-labeled cells induced at E10.5 has demonstrated that, although these progenitors are initially located outside the condensation at E11.5, they invade the cartilage anlage by E13.5 [26,36]. From birth, Hes1^+^ descendants differentiate into chondrocytes, cortical and trabecular osteoblasts, and bone marrow stromal cells (BMSCs), with lineage allocation governed by chondrogenic SOX9 programs, osteogenic RUNX2/SP7 hierarchies, and stromal-associated transcriptional states; by postnatal day 21 (P21), they contribute broadly to all skeletal lineages [26] (Figure 1). Asymmetric division of Hes1^+^ progenitors may underlie this versatility: One daughter cell retains Hes1 and remains an undifferentiated perichondrial stem cell, whereas the other downregulates Hes1 expression, enters the Sox9^+^ domain, and commits to chondrogenesis [26,37,38].

By E10.5–11.5, Fgfr3 expression becomes restricted to a subset of Sox9-positive condensations, particularly localized to the central region at E11.5, marking the chondroprogenitors committed to proliferative expansion [16]. Lineage labeling at E10.5 using *Fgfr3-CreER*; *R26R*^tdTomato^ embryos has revealed that, by E13.5, these cells are located almost exclusively in the dorsal resting zone of the cartilage anlage, with minimal contribution to ventral or proliferative regions [16]. Their descendants persist in the postnatal skeleton and contribute to dorsal metaphyseal chondrocytes, cortical osteoblasts, and BMSCs. Notably, this dorsal bias extends to other appendicular elements but is absent in vertebral bones (Figure 1).

At E10.5, mesenchymal condensation segregates into distinct progenitor compartments: Fgfr3^+^ chondroprogenitors, which drive cartilage anlage expansion with a dorsal bias, are located centrally and Hes1^+^ cells, which refine condensation boundaries and contribute to both cartilage and perichondrial lineages, are positioned peripherally [16,26]. The coordinated activation and repression of Prrx1, Sox9, Dermo1, Pdgfra, Hes1, and Fgfr3 ensure that endochondral ossification proceeds with precise spatial and temporal patterning, laying the blueprint for adult skeletal architecture.

## 3. Fates of Skeletal Progenitor Cells During the Cartilage Anlage Stage

By E13.5, mesenchymal condensation matures into a defined cartilage anlage, and its constituent progenitors begin to adopt more specialized trajectories [39]. Within the anlage, Sox9^+^-lineage cells labeled at E10.5 (Sox9^CE^-E10.5) almost completely overlap with Sox9^+^ chondrocytes and contribute broadly to the formation of resting, proliferative, and hypertrophic zones [16]. These zones are established by distinct differentiation programs: Sox9 maintains chondrocyte identity in the resting zone, the Ihh–PTHrP feedback loop sustains proliferative expansion and regulates the pace of hypertrophic entry, and Runx2 drives hypertrophic differentiation. When labeling is deferred until E12.5 (Sox9^CE^-E12.5), these descendants account for approximately 90% of chondrocytes but only approximately half of perichondrial cells. Dorsoventral bias is not observed in either case: Sox9^+^ cells are distributed equally across dorsal and ventral compartments, underscoring their fundamental role in cartilage anlage expansion [16]. Notably, the short-chase expression pattern of *Sox9-CreER* is broader than that observed via SOX9 immunohistostaining. However, whether *Sox9-CreER* targets a wider range of cell types, including chondrocytes and perichondrial cells, and whether Sox9^+^ chondrocytes contribute to outer perichondrial cells remains unclear.

Perichondrial skeletal progenitors emerge around the cartilage anlage in two distinct waves between E12.5 and E13.5. First, Sp7 (Osx) is expressed in the inner perichondrial layer, marking a population of transient osteogenic precursors [20]. Inner perichondrial cells labeled by *Osx-CreER* at E12.5 mainly give rise to trabecular osteoblasts and BMSCs at birth. However, most descendants disappear by P21 [20]. In contrast, Dlx5 expression localizes to the outer perichondrial layer during the cartilage anlage stage. This outer perichondrial layer represents a critical osteogenic compartment that serves as a progenitor pool for subsequent periosteum formation and bone collar development. *Dlx5-CreER*-labeled cells at E12.5 contribute robustly to periosteal cells, the bone collar, trabecular osteoblasts, and BMSCs at birth. Through these contributions, Dlx5^+^ outer perichondrial progenitors play an essential role in initiating perichondrial ossification and establishing the cortical bone template of the diaphysis. Their descendants are largely restricted to diaphyseal skeletal cells by P21 [20]. Although single-cell RNA-sequencing data have indicated low *Dlx5* expression at E11.5, fate mapping has shown that *Dlx5-CreER* cells in the condensation stage contribute to the dorsal side of the outer-layer perichondrial cells surrounding the cartilage anlage [16,20]. These dorsal *Dlx5*-*CreER* progenitors form the bone collar and ultimately the diaphyseal cortex, maintaining a dorsal-focused distribution through P7 and P21 [16] (Figure 1).

In addition to Osx^+^ and Dlx5^+^ perichondrial waves, hedgehog-responsive mesenchymal cells marked by *Gli1-CreER* contribute broadly to cartilage and bone lineages. Shi et al. reported that, at E14.5, *Gli1-CreER*-labeled cells contribute to skeletal elements predominantly in the perichondrium and, to a lesser extent, in the cartilage in the adult stage [22].

*Fgfr3-CreER*-labeled chondroprogenitor cells remain restricted to the center of the cartilage anlage at E13.5 [16,20]. Postnatally, these descendant cells give rise to metaphyseal stromal cells distinct from *Dlx5*-*CreER*-derived diaphyseal BMSCs [20]. Transcriptional profiling of these two fetal-origin BMSC pools has revealed functional divergence: *Fgfr3*-*CreER*-derived BMSCs express high levels of osteogenic markers (*Acan*, *Col2a1*, *Alpl*, and *Sp7*) and respond robustly to PTH-driven bone formation, whereas *Dlx5*-*CreER*-derived BMSCs upregulate adipogenic marker (*Adipoq*) levels and exhibit minimal PTH responsiveness [20,39,40].

In a bone marrow ablation model designed to mimic injury-induced osteogenesis, *Dlx5*-*CreER*-derived BMSCs contributed predominantly to diaphyseal regeneration, whereas *Fgfr3*-*CreER*-derived BMSCs drove repair in the metaphysis [20]. These results suggest that adipogenic vs. osteogenic BMSC lineages arise from spatially and molecularly distinct embryonic progenitors: Dlx5^+^ fetal perichondrial cells seed diaphyseal adipocyte-biased stromal niches, whereas Fgfr3^+^ fetal chondrocytes establish a metaphyseal osteoblast-biased microenvironment. In addition to these embryonically defined perichondrial- and cartilage anlage-derived stromal lineages, lineage-tracing studies have demonstrated that resting and hypertrophic chondrocytes can directly contribute to osteoblasts, osteocytes, and stromal cells during endochondral ossification [13,19,41]. This cartilage-derived contribution represents an additional cellular connection between the cartilage anlage and nascent bone formation.

## 4. Signaling Networks Governing Growth Plate Patterning

**Figure 2:**
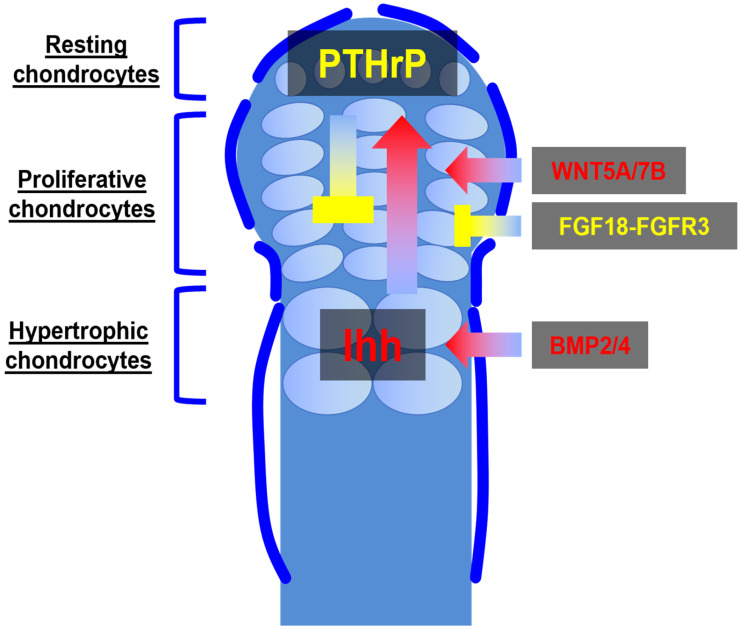
Hierarchical signaling network regulating growth-plate organization. Schematic of the trilaminar growth plate showing resting, proliferative, and hypertrophic zones. Ihh–PTHrP axis: Ihh from hypertrophic chondrocytes induces PTHrP in periarticular and resting zone cells; PTHrP acts on proliferative chondrocytes to delay hypertrophy and preserve the proliferative pool. FGF18–FGFR3 axis: FGF18 produced in the perichondrium signals via FGFR3 on proliferative chondrocytes to slow cell-cycle progression and restrict proliferative expansion. BMP2/4: BMP2 and BMP4 concentrate in the prehypertrophic/hypertrophic region to activate SMAD1/5/8, promote Runx2 and Col10a1 expression, and drive terminal hypertrophy. WNT5A/7B: Noncanonical WNT signaling, exemplified by WNT5A and potentially involving WNT7B in certain contexts, regulates planar cell polarity and columnar alignment in the proliferative zone, whereas canonical WNT signaling acts through β-catenin downstream of LRP5/6 receptors at the chondrocyte–perichondrial interface to promote osteogenic differentiation. Different colors indicate distinct growth plate zones and signaling domains. Red arrows denote activating effects, whereas yellow T-bars indicate inhibitory effects.

After the establishment of the cartilage anlage by E12.5, the developing anlage acquires a highly ordered trilaminar structure by approximately E15.5, in which chondrocytes progress from a quiescent “resting” state through proliferative expansion to terminal hypertrophy [42]. This zonal organization is orchestrated by a network of interdependent signaling pathways, chief among them the Ihh–PTHrP, FGF, bone morphogenetic protein (BMP), and WNT/β-catenin pathways, which collectively balance chondrocyte proliferation, differentiation, and apoptosis [43,44]. Notably, RUNX2 is a key downstream target of BMP and WNT/β-catenin signaling at the chondro-osseous junction, promoting hypertrophic maturation and osteogenic programs [45,46] (Figure 2).

The Ihh–PTHrP feedback loop lies at the apex of this regulatory hierarchy. Hypertrophic chondrocytes in the lower zone secrete Ihh, which diffuses upward to stimulate PTHrP expression in periarticular and resting zones [47]. Subsequently, PTHrP binds to its receptor (PTH1R) on proliferative chondrocytes to delay the onset of hypertrophy, thereby expanding the proliferative pool [48]. As cells divide and are displaced away from the PTHrP source, they lose this inhibitory signal, allowing Ihh to induce their transition to a hypertrophic phenotype characterized by Col10a1 expression and matrix mineralization [49]. Ablation of Ihh or PTHrP in mice disrupts this delicate equilibrium: Ihh deficiency accelerates hypertrophy and prematurely fuses the growth plate, whereas loss of PTHrP leads to depleted proliferative chondrocytes and shortened bones [50,51]. Notably, Ihh not only reinforces chondrocyte proliferation indirectly via PTHrP but also promotes osteoblast lineage commitment in the adjacent perichondrium, linking growth plate dynamics to bone collar formation [52].

FGF signaling acts as a second tier of control, restraining chondrocyte proliferation and coordinating the pace of hypertrophic progression [53]. Among the multiple FGF ligands expressed in the perichondrium, FGF18 is particularly critical [54,55]. It binds to Fgfr3 on proliferating chondrocytes and activates downstream mitogen-activated protein kinase and signal transducer and activator of transcription-1 cascades that suppress cyclin D1 expression, thereby limiting cell cycle entry [56,57]. Mice harboring activating *Fgfr3* mutations as models of achondroplasia exhibit an abnormally narrow proliferative zone and accelerated hypertrophy, emphasizing the role of Fgfr3 as a brake on growth plate expansion [58,59]. Conversely, *Fgfr3* knockout animals exhibit an expanded proliferative compartment and delayed differentiation [60]. Therefore, FGFR3 signaling possibly regulates the Ihh–PTHrP axis by controlling the number of cells available for Ihh production and their responsiveness to PTHrP.

BMPs constitute another critical layer in growth plate regulation, performing dual functions depending on dosage and spatial context [61]. BMP2 and BMP4 are expressed in the prehypertrophic region, where they synergize with Ihh to promote chondrocyte hypertrophy [62,63,64]. BMP signaling via SMAD1/5/8 enhances the expression of Runx2 and Col10a1, accelerating terminal differentiation [65,66,67]. Simultaneously, BMPs induce Sox9 expression in the resting zone, supporting early chondrogenesis [68]. Disruption of BMP receptors in chondrocytes leads to growth plate disorganization, underscoring the indispensable and context-dependent role of BMP in coordinating proliferation and hypertrophy [69].

WNT/β-catenin signaling intersects with the Ihh, FGF, and BMP pathways to refine growth plate architecture. Canonical WNT ligands (e.g., WNT3A) are predominantly active at the chondrocyte–perichondrial interface, where WNT/β-catenin signaling suppresses *Sox9* expression and promotes the osteogenic differentiation of perichondrial cells [70,71,72]. Within the growth plate, non-canonical WNTs, such as WNT5A and WNT7B, contribute to planar cell polarity, aligning columns of proliferative chondrocytes to ensure orderly columnar growth [73,74]. Loss of Wnt5a disrupts columnar organization, producing a disorganized growth plate with reduced proliferation [44]. Therefore, distinct branches of the WNT family either reinforce hypertrophy and perichondrial ossification or maintain the structural integrity of proliferative columns.

Extracellular factors such as vascular endothelial growth factor (VEGF) and matrix metalloproteinases (MMPs) facilitate the transition from chondrocyte hypertrophy to ossification [75,76,77]. Hypertrophic chondrocytes secrete VEGF, which stimulates endothelial invasion of the calcified cartilage matrix [78]. This process occurs at the chondro-osseous junction, where terminal hypertrophic chondrocytes define a discrete zone of calcified cartilage that serves as a transient mineralized scaffold [79]. Adjacent to this region, the cartilage erosion front is established by invading endothelial cells, osteoclasts, and osteoprogenitors, enabling coordinated cartilage resorption and replacement by bone. Concurrently, MMP13 produced by hypertrophic chondrocytes mediates degradation of the cartilage extracellular matrix, thereby facilitating osteoclast-mediated resorption and osteoprogenitor invasion [80,81]. Collectively, these events culminate in the replacement of hypertrophic cartilage by trabecular bone [81,82].

Together, these interconnected signaling circuits establish a self-regulating network, in which feedback loops, gradient formation, and cross-pathway interactions precisely dictate growth plate zonation. By coupling proliferative expansion to regulated hypertrophy and vascular invasion, this system ensures the proportional elongation of long bones while preparing the scaffold for timely ossification.

## 5. Mechanisms of Dorsoventral Patterning in the Limb Bud

Dorsoventral polarity is established in the limb field well before mesenchymal condensation occurs. In mice, LIM homeobox transcription factor-1 beta (Lmx1b) expression is first detectable in the lateral plate mesoderm at the presumptive forelimb level as early as E8.5, shortly thereafter appearing in the hind limb anlage [83]. By E9.5–10.0, Lmx1b becomes confined to the dorsal half of the emerging limb bud mesenchyme, marking the first molecular manifestation of dorsoventral identity [83]. This early Lmx1b domain is induced by Wnt family member 7A (Wnt7a) secreted from the overlying dorsal ectoderm. Genetic loss of Wnt7a abolishes Lmx1b expression and dorsal limb characteristics, whereas ectopic Wnt7a imposes dorsal fate on ventral tissues [84,85]. Complementing this, engrailed homeobox 1 (En1) is expressed in the ventral ectoderm and represses Wnt7a ventrally, thereby sharpening the dorsal field and reinforcing ventral identity before cartilage condensation [83,85].

After the establishment of this broad dorsoventral framework, two key progenitor populations emerge exclusively within the specified dorsal domain. Fgfr3-expressing cells localize centrally within the cartilage anlage and, upon lineage-marking at E10.5, give rise to dorsal growth plate chondrocytes, cortical osteoblasts, and BMSCs throughout development [16]. Surrounding them, Dlx5^+^ cells occupy the outer perichondrial layer and subsequently form the dorsal bone collar and diaphyseal cortex [16]. In comparison, the ventral compartment harbors its own lineage restricted progenitors: Molecules such as Tbx18 and tenascin-C, present at the ventral rim of condensation, possibly mark ventral chondrogenic or osteogenic precursors [16], although their definitive roles remain unclear.

The dorsoventral axis of the limb bud emerges in two temporally distinct phases: First, Wnt7a–Lmx1b and En1 establish broad dorsal vs. ventral competence in the mesenchyme by E9.5; then, during mesenchymal condensation at E10.5, Fgfr3 and Dlx5 selectively demarcate chondrogenic and osteogenic progenitors within the dorsal domain. This sequential patterning ensures that skeletal cell lineages inherit the correct positional identity well before ossification. Emerging evidence further suggests that this early dorsoventral specification may be stabilized by epigenetic mechanisms, including enhancer priming and lineage-restricted chromatin accessibility, which reinforce positional identity once progenitor fates are established [86,87].

## 6. Insights into Congenital Skeletal Disorders

Congenital skeletal malformations often trace back to perturbations in the tightly regulated sequence of events shaping the embryonic limb bud. Here, we discuss selected examples that illustrate how disruption of specific developmental nodes leads to defined skeletal phenotypes, from mesenchymal condensation through cartilage anlage formation and perichondrial patterning. Achondroplasia, the most common form of dwarfism, arises from the constitutive activation of *FGFR3* (e.g.,*G380R* mutation), which transiently restrains the proliferation of dorsal resting chondrocytes to modulate growth during normal development [88]. When FGFR3 signaling becomes ligand-independent, proliferative chondrocytes exit the cell cycle prematurely and undergo accelerated hypertrophy, collapsing the proliferative zone and producing characteristic rhizomelic shortening [89]. In mouse models, this defect is most pronounced in the dorsal metaphysis, reflecting the spatial restriction of Fgfr3^+^ progenitors to that region. Conditional ablation of these cells similarly impairs dorsal bone formation, underscoring the locus of pathology [16].

Campomelic dysplasia exemplifies the mechanism by which *SOX9* haploinsufficiency disrupts early skeletal patterning [90]. During normal development, *Sox9* expression begins during mesenchymal condensation (around E10.5), where it directly activates key cartilage matrix genes (e.g., *Col2a1* and *Acan*) and maintains chondrocyte progenitor identity [9]. Heterozygous loss-of-function mutations or regulatory deletions in *Sox9* abolish its role in initiating chondrogenic condensation and maintaining chondrocyte progenitor identity, resulting in insufficient condensation, markedly reduced *Col2a1* and *Acan* expression, and impaired growth plate organization [91,92]. In *Sox9*^+/−^ embryos, condensations are hypoplastic at E12.5, with delayed mesenchymal–chondrocyte transition, and long bones (e.g., radius, ulna, and tibia) and scapulae exhibit pronounced bending and hypoplasia by E14.5 [91,92]. Subsequent expansion of the hypertrophic zone and premature mineralization reflect an inability to suppress chondrocyte hypertrophy [91]. Clinically, affected infants present with bent long bones, scapular hypoplasia, and often lethal respiratory compromise [91]. Histologically, growth plates are disorganized, condensations remain mesenchymal, and chondroblast differentiation is delayed despite preserved proliferation rates, indicating that *Sox9* dosage is essential in both the condensation and growth plate maturation stages [93].

Split-hand/foot malformation (SHFM) type I illustrates the effects of *DLX5* haploinsufficiency on skeletal patterning [94]. In patients with SHFM-I, heterozygous deletions or loss-of-function mutations affecting the *DLX5*/*DLX6* cluster result in the absence or hypoplasia of the central digital rays, often accompanied by cortical thinning of the metacarpals and metatarsals [95]. Mouse models harboring a targeted *Dlx5* null allele recapitulate key features, with limb buds exhibiting reduced proliferation in the apical ectodermal ridge and expanded cell death in the dorsal mesenchyme, leading to split autopods and dorsal skeletal hypoplasia [96]. Histological analysis has revealed that, despite normal early condensation, the dorsal perichondrial compartment fails to form a robust bone collar and that chondrocyte hypertrophy is prematurely initiated, producing malformed cortical bone [95]. Affected mice also exhibit craniofacial defects, underscoring the broader role of *DLX5* in osteogenic progenitor maintenance [97].

Cleidocranial dysplasia further underscores the central role of osteogenic transcriptional control in skeletal development. This disorder is caused by haploinsufficiency of RUNX2, a master regulator of osteoblast differentiation downstream of BMP and WNT/β-catenin signaling. Reduced RUNX2 dosage impairs bone collar formation, delays intramembranous ossification, and compromises cortical bone integrity, leading to characteristic hypoplastic clavicles, delayed cranial suture closure, and generalized skeletal dysplasia [98,99,100].

In addition to patterning defects driven by lineage-specific transcription factors, perturbations of systemic and local signaling pathways also give rise to diffuse growth plate dysplasias. Congenital forms of rickets caused by mutations in PHEX, FGF23, or DMP1 exemplify how altered phosphate homeostasis and FGF signaling impair growth plate mineralization and endochondral ossification [101,102]. In Jansen metaphyseal chondrodysplasia, *PTH1R* mutations prevent chondrocytes from escaping PTHrP-mediated inhibition, elongating the proliferative zone and delaying hypertrophy [103], whereas *IHH* mutations in brachydactyly type A1 cut short the feedback loop, resulting in premature hypertrophy and shortened digits [104]. These reports highlight the importance of precise spatiotemporal coordination of signaling pathways for normal growth plate zonation.

## 7. Emerging Bone Regeneration Strategies

Recent insights into embryonic endochondral ossification have not only advanced our understanding of skeletal development but have also opened new avenues for clinically relevant strategies aimed at cartilage regeneration and the repair of large, critical-sized bone defects. In particular, recognition of the distinct and coordinated contributions of chondroprogenitors and osteoprogenitors has inspired regenerative approaches that deliberately recapitulate developmental programs to overcome the limitations of conventional bone grafts and scaffold-based therapies. One key advancement is the design of “ossification center-like organoids” (OCOs), which combine a mesenchymal stem cell (MSC)- and BMP-2-laden osteogenic core with substance P, known for recruiting endogenous MSCs to local injuries [105]. In large bone defects, OCOs accelerate bridging by recruiting Krt8^+^ skeletal stem cells (SSCs) and limiting fibrotic Has1^+^ fibroblasts, thereby recreating a pro-regenerative niche reminiscent of fetal bone formation [105]. Similarly, “osteo-callus organoids” assembled in three-dimensional (3D)-printed gelatin methacryloyl (GelMA) microspheres complete endochondral ossification within four weeks in rabbit models, outpacing conventional repair timelines by nearly two months [106].

Simultaneously, progress in biomaterials has yielded scaffolds with precisely tuned spatiotemporal signaling. Collagen hydrogels co-delivering costal cartilage, derived stem cells, and the VEGF-binding peptide PR1P enhance endogenous VEGF levels by over three-fold, promote osteogenic differentiation, and reduce osteoclast activity by nearly 70%, achieving coordinated vascularization, bone formation, and anti-resorptive effects [107]. Composite scaffolds, such as epigallocatechin gallate (EGCG)-modified gelatin sponges, improve hydrophilicity and foster calcium phosphate deposition, driving robust osteogenesis in craniofacial defect models [108]. For critical-sized bone defects, researchers have developed inorganic–organic multifunctional composite hydrogels called poly(L-glutamic acid)-g-tyramine (PLG-g-TA)/VEGF/Sr-containing bioactive glass nanoparticles (Sr-BGNPs), which simultaneously enhance BMSC proliferation, migration, and osteogenic differentiation, facilitating in situ bone regeneration [109].

Stem cell-based interventions are also continuously advancing. In pediatric osteogenesis imperfecta, systemic infusion of allogeneic bone marrow-derived MSCs leads to engraftment within the bone and cartilage, increased growth velocity, enhanced bone mineral content, and reduced fracture rates over follow-up periods of up to two years [110]. Compared to BMSC-derived constructs, autologous induced pluripotent stem cell (iPSC)-derived MSC chondrocytes delivered in fibrin/nanofiber scaffolds generate superior hyaline-like cartilage in porcine femoral condyle defects, as indicated by elevated *COL2A1* levels [111]. Exosome mimetics produced by extrusion from human MSCs enriched in CD63-positive vesicles and further enhanced by noggin knockdown promote robust bone regeneration via miR-29a-mediated osteogenic pathways in mouse calvarial defects when delivered in a chitosan hydrogel [112]. Taken together, these innovations highlight the growing potential of stem cell-based approaches to regenerate complex skeletal tissues.

Despite these promising avenues, several challenges remain to be addressed. Broad clinical application of autologous or allogeneic cell therapies requires rigorous evaluation of their long-term safety, including potential inflammatory responses to implanted scaffolds, host rejection, and immune reactions. The high costs associated with many novel treatments also need to be addressed. Resolving these issues will enhance the clinical translation of regenerative bone therapies, offering new hope to patients with congenital defects, traumatic injuries, and degenerative bone diseases.

## 8. Conclusions

Over the past decade, our understanding of endochondral ossification has progressed from a simple linear model of cartilage anlage formation to an integrated multidimensional framework of tightly orchestrated cell fate decisions. Mesenchymal condensation at E10.5 does not generate a homogeneous progenitor pool but instead rapidly segregates into distinct lineages: Centrally located Sox9^+^/Fgfr3^+^ chondroprogenitors, which expand into the dorsal growth plate and metaphyseal stroma, and peripheral Dlx5^+^ osteoprogenitors, which give rise to the bone collar and diaphyseal cortex [16,20]. Concurrently, Hes1*^+^* boundary cells refine this aggregate via asymmetric division, seeding both the cartilage and perichondrial compartments [26]. These early divergences establish the blueprint for lifelong skeletal architecture and explain why mutations in regulators such as *FGFR3*, *SOX9*, and *DLX5* lead to region-specific dysplasias, including achondroplasia, campomelic dysplasia, and SHFM. At the osteochondral interface, RUNX2 acts downstream of these early lineage decisions to drive chondrocyte hypertrophy, matrix remodeling, and osteoblast differentiation, thereby coupling growth plate maturation to bone collar and cortical bone formation [113,114].

Superimposed on these lineage bifurcations is a network of interacting signaling pathways, including Ihh–PTHrP, FGF, BMP, and WNT/β-catenin pathways, which collectively impose temporal control over chondrocyte proliferation, hypertrophy, and vascular invasion. Disruption of any node within this feedback circuit results in growth plate disorders, ranging from Jansen metaphyseal chondrodysplasia to brachydactyly. Furthermore, dorsoventral polarity, established by Wnt7a–Lmx1b and En-1 before condensation, becomes epigenetically locked, ensuring that dorsal Fgfr3^+^ and Dlx5^+^ progenitors maintain their positional identity into adulthood.

Importantly, the developmental principles outlined here are not restricted to the appendicular skeleton but also bear relevance to craniofacial skeletal elements that undergo endochondral ossification. Structures such as the mandibular condyle and cranial base synchondroses employ conserved regulatory modules, including SOX9-dependent mesenchymal condensation and Ihh–PTHrP-, FGF-, BMP-, and WNT-mediated control of chondrocyte maturation and growth plate organization [79,115]. Nevertheless, in contrast to mesoderm-derived limb and axial bones, craniofacial endochondral structures largely originate from neural crest cells and exhibit distinct growth plate architectures, prolonged postnatal plasticity, and heightened sensitivity to biomechanical cues [116,117]. These shared mechanisms and region-specific adaptations suggest that early progenitor partitioning provides a transferable yet context-dependent framework for understanding craniofacial skeletal development and disease.

By harnessing the lessons encoded in embryonic development, researchers can engineer bone tissues with remarkable precision. 3D organoid cultures faithfully reproduce the key features of ossification centers, and advanced hydrogels release morphogens in space and time to guide cell differentiation. Moreover, stem cell- and exosome-based delivery platforms leverage developmental miRNA networks to regenerate region-specific skeletal tissues.

In summary, the sequence of embryonic events governing mesenchymal condensation and dorsoventral patterning offers both a roadmap to unravel skeletal disorders and a blueprint to design next-generation regenerative therapies. As our understanding of developmental signaling becomes more precise, engineering fully functional bone tissues shifts from a distant aspiration to a tangible reality.

## Data Availability

No new data were created or analyzed in this study. Data sharing is not applicable to this article.

## Abbreviations

The following abbreviations are used in this manuscript:

Acan	Aggrecan
BMP	Bone morphogenetic protein
BMSCs	Bone marrow stromal cells
Dlx5	Distal-less homeobox 5
En1	Engrailed homeobox 1
Fgfr	Fibroblast growth factor receptor
Gli1	GLI family zinc finger 1
Hes1	Hes family bHLH transcription factor 1
Ihh	Indian hedgehog
Lmx1b	LIM homeobox transcription factor-1 beta
MMPs	Matrix metalloproteinases
Osx	Osterix
Pdgfra	Platelet-derived growth factor receptor-alpha
Prrx1	Paired-related homeobox 1
PTHrP	Parathyroid hormone-related protein
Runx2	Runt-related transcription factor 2
SHFM	Split-hand/foot malformation
Sox9	Sry-box transcription factor 9
VEGF	Vascular endothelial growth factor
Wnt7a	Wnt family member 7A
